# Cadmium Dose-Responsive Transcriptomics Prioritizes *CvHMA15* and *CvHMA16* in *Cardamine violifolia*

**DOI:** 10.3390/plants15213251

**Published:** 2026-10-22

**Authors:** Hu Gao, Xingxue Huang, Haixiao Wang, Anhua Ye, Yangyi Li, Jiajia Ming, Bingye Gu, Hongqing Yin, Guolin Zhou, Runhua Zhang

**Affiliations:** 1 Institute of Vegetable, Wuhan Academy of Agricultural Sciences, Wuhan 430045, China; 2 Enshi Tujia and Miao Autonomous Prefecture Academy of Agricultural Sciences, Enshi 445000, China

**Keywords:** *Cardamine violifolia*, cadmium stress, dose-responsive transcriptomics, heavy metal ATPase, yeast functional assay

## Abstract

Cadmium (Cd) responses in metal-accumulating plants involve coordinated stress programs and specialized metal transporters, but the relationship between these levels remains poorly resolved in *Cardamine violifolia*. Here, seeds were germinated for seven days under 0, 10, 25, or 100 mg L^−1^ CdCl_2_, followed by root phenotyping and dose-resolved transcriptome analysis. Root growth showed a biphasic pattern: root length increased at 10 mg L^−1^, whereas growth at 100 mg L^−1^ was strongly reduced relative to the low-dose treatment. RNA-seq of 12 libraries identified 5665 genes that responded in at least one comparison and resolved six expression trajectories. Pathway analysis indicated low-dose metabolic adjustment, strong activation of phenylpropanoid, signaling, and xenobiotic-response pathways at 25 mg L^−1^, and broader transcriptional repression at 100 mg L^−1^. Cross-referencing the transcriptome with 16 reference-identified CvHMA loci prioritized *CvHMA15* and *CvHMA16* as a 99.87 identical protein pair with divergent regulation: *CvHMA15* was moderately down-regulated across the Cd gradient, whereas *CvHMA16* was induced and met the combined differential-expression threshold at 100 mg L^−1^. In a pYES2.0-based yeast spot assay, expression of either *CvHMA15* or *CvHMA16* maintained colony growth across five cell densities on medium containing 50 μM CdCl_2_, whereas the empty-vector control was strongly inhibited; growth was comparable among transformants in the absence of Cd. These results establish a transcriptome-guided framework for candidate prioritization while leaving transport direction and in planta mechanism to direct functional tests.

## 1. Introduction

Heavy metal contamination in agricultural soils has become a major environmental concern, threatening both ecosystem stability and human health [1]. High concentrations of heavy metals can severely impair plant growth, development, and yield by damaging cellular structures and membranes, inducing excessive accumulation of reactive oxygen species (ROS), and disrupting metabolic processes and nutrient transport. Similar physiological responses have also been observed under other abiotic stresses such as drought and salinity [2]. Hyperaccumulator plants are capable of absorbing and sequestering exceptionally high levels of heavy metals, making them effective tools for phytoremediation. These species maintain physiological homeostasis through tightly regulated metal uptake, chelation or compartmentalization of metal ions within cellular organelles, and activation of antioxidant enzyme systems to alleviate oxidative stress [3]. Consequently, the use of hyperaccumulators represents an environmentally friendly and cost-effective strategy for mitigating heavy metal pollution.

Cadmium (Cd), a non-essential and highly toxic heavy metal, has emerged as a significant environmental pollutant, particularly in soils affected by intensive agricultural and industrial activities [4]. Excessive Cd accumulation in plants leads to overproduction of ROS, causing leaf chlorosis, growth inhibition, and even cell death. To counteract Cd-induced oxidative stress, plants rely on complex enzymatic and non-enzymatic antioxidant systems to maintain cellular redox balance and homeostasis [5]. Additionally, Cd can interfere with chlorophyll synthesis by partially replacing magnesium ions, resulting in reduced chlorophyll content and impaired photosynthetic capacity [6]. Beyond plant toxicity, Cd poses a threat to human health through bioaccumulation in the food chain, highlighting the importance of understanding Cd uptake, translocation, and tolerance mechanisms for food safety and environmental protection [7].

Heavy metal ATPases (HMAs), also known as P1B-type ATPases, are membrane-bound transporters that play central roles in metal ion transport and homeostasis in plants [8]. Genome-wide and molecular studies have characterized HMA gene families in several plant species, revealing two main subclasses based on metal substrate specificity: the Cu/Ag group and the Zn/Co/Cd/Pb group [9,10,11]. Nevertheless, individual HMA functions differ among species. *AtHMA3* promotes vacuolar sequestration of Cd, Zn, Co, and Pb in *Arabidopsis thaliana* [12], whereas *SpHMA3* contributes to Cd hypertolerance in *Sedum plumbizincicola* [13]. Cd-responsive transporters identified by transcriptome analysis in *Cucumis sativus* further illustrate the value of interpreting transporter candidates within the wider stress-response network [14]. Copy-number expansion and elevated expression of *HMA4* have been associated with metal adaptation in *Arabidopsis halleri* and *Noccaea caerulescens* [15,16,17]. These observations make HMA diversification a plausible component of metal adaptation, but neither phylogenetic position nor expression pattern alone establishes substrate specificity, membrane localization, or transport direction.

*Cardamine violifolia*, a Brassicaceae species native to the Enshi region of China, is recognized for exceptional selenium accumulation [18,19,20], and physiological studies have also reported substantial Cd accumulation [21]. The chromosome-level assembly used in this study was published under the name *Cardamine enshiensis* and contains 16 pseudomolecules (2n = 32; 443.4 Mb) [22]. This genome provides the reference for CvHMA identification, but the dose-dependent transcriptomic response to Cd and the functional prioritization of individual *CvHMA* genes have not been integrated in one experimental framework. We therefore characterized root responses to a CdCl_2_ gradient and performed whole-transcriptome differential-expression, trajectory, and pathway analyses. The transcriptomic dataset was then cross-referenced with reference-annotated CvHMA sequences to prioritize candidates, after which phylogenetic, structural, comparative genomic, and tissue-expression analyses provided evolutionary and expression context. Finally, the closely related but transcriptionally divergent *CvHMA15*/*CvHMA16* pair was tested in a pYES2.0 yeast assay. This design distinguishes direct functional evidence from hypotheses about transport direction and in planta mechanism.

## 2. Results

### 2.1. Biphasic Root Response of Cardamine violifolia to CdCl_2_

**Figure 1:**
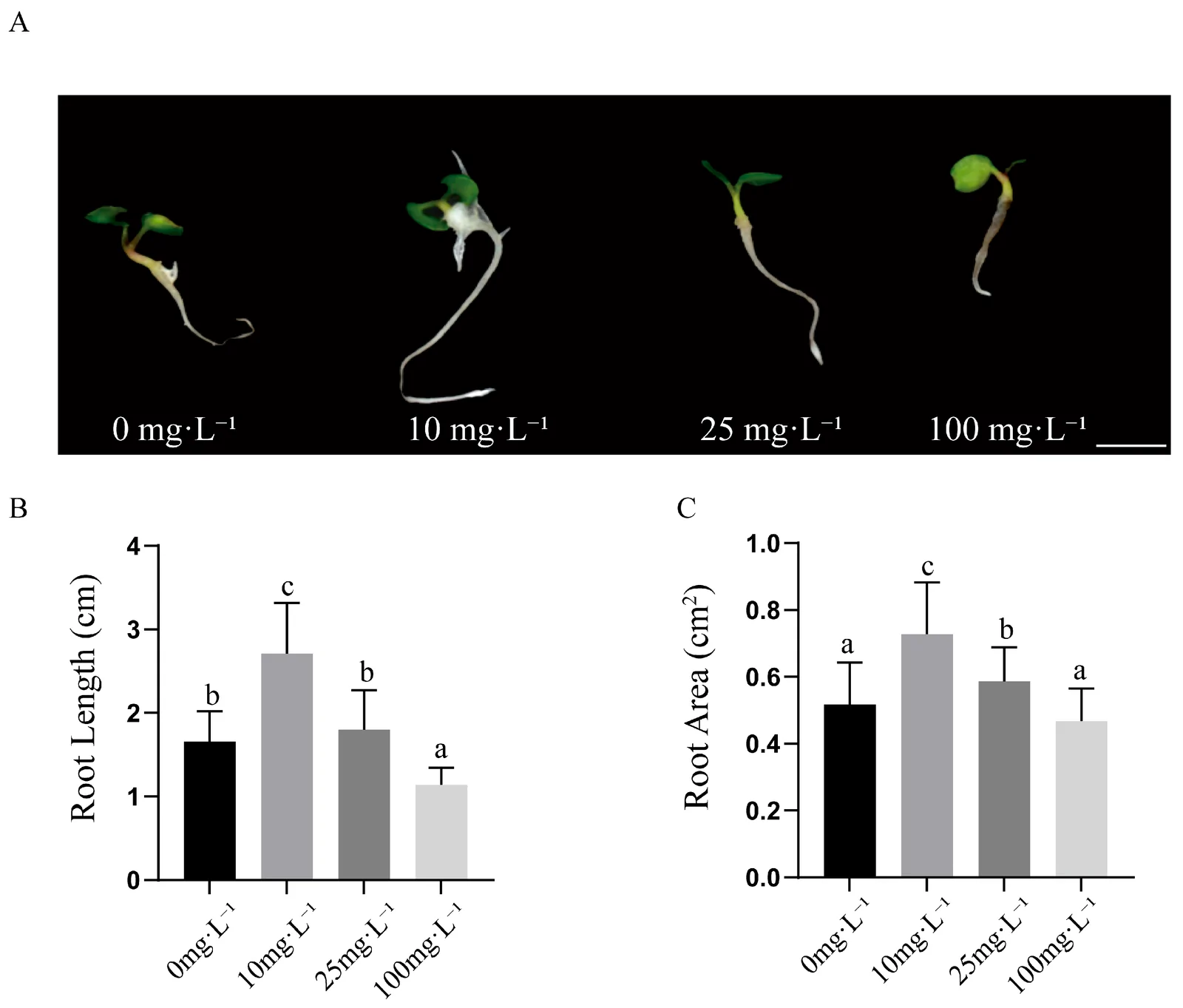
Effects of CdCl_2_ exposure on seedling root development in *Cardamine violifolia*. (**A**) Phenotypes of seedlings under 0, 10, 25, and 100 mg L^−1^ Cd treatments, photographed 7 days after treatment. Scale bar = 5 mm. Statistical analysis of root length (**B**) and root area (**C**). Data are presented as mean ± SD (*n* = 3). Different lowercase letters indicate significant differences among treatments (*p* < 0.05).

Phenotypic analysis revealed a biphasic dose–response relationship in *Cardamine violifolia* seedlings under varying Cd concentrations (Figure 1). Treatment with 10 mg·L^−1^ Cd significantly increased root length and root surface area by approximately 1.5-fold and 1.3-fold, respectively, compared to the control (Figure 1B,C). At the moderate concentration of 25 mg·L^−1^, no statistically significant reduction in root length was observed, while root surface area remained significantly higher than that of the control. Significant growth inhibition occurred only at 100 mg·L^−1^, where root length decreased to approximately 70% of the control value. This reduction was accompanied by visible browning of non-apical mature root regions, whereas the root tips remained visibly white (Figure 1A).

### 2.2. Cd Dose Gradient Drives Global Transcriptional Remodeling and Forms Six Expression Response Trajectories

**Figure 2:**
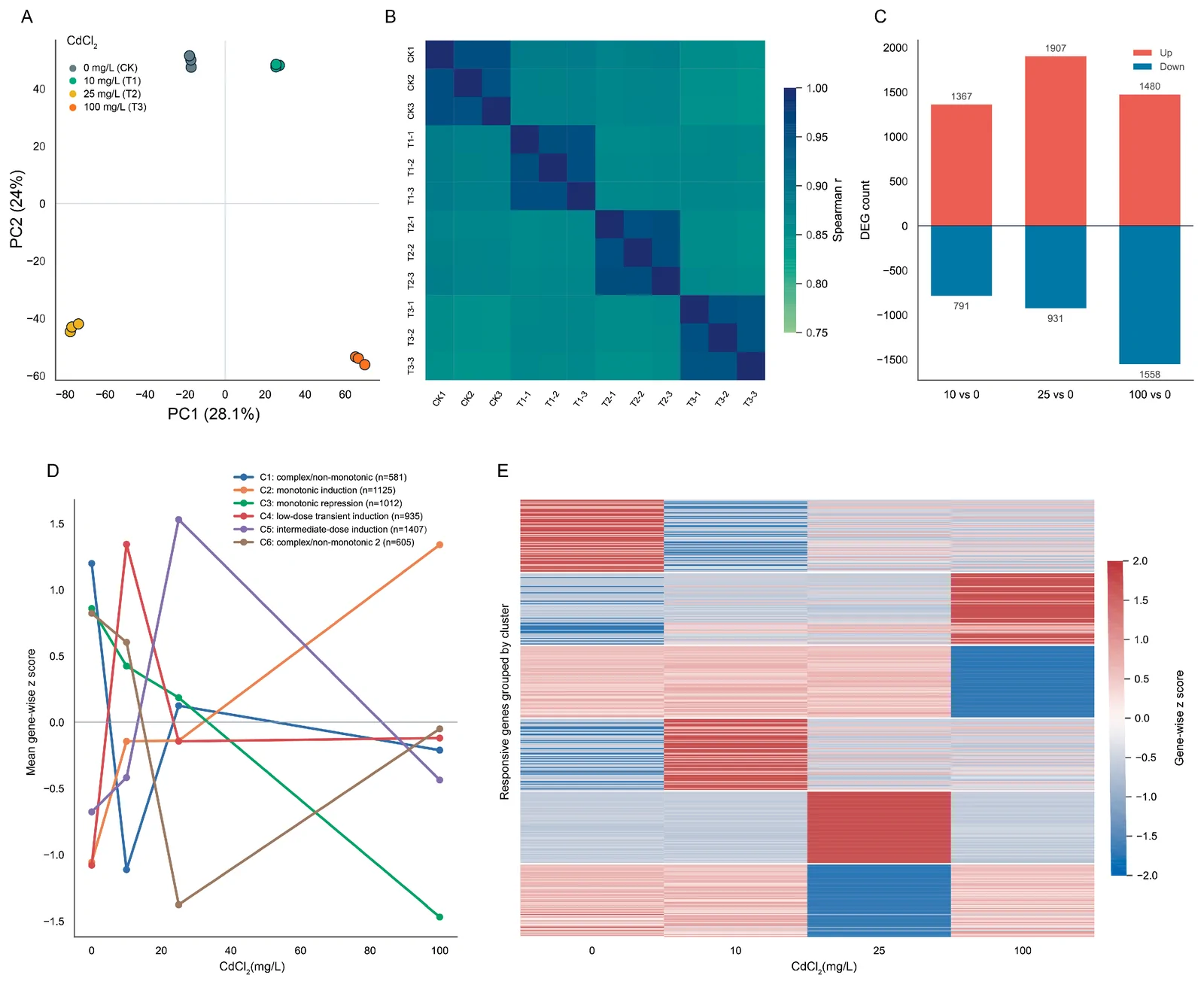
Global transcriptome response to a CdCl_2_ gradient (**A**) PCA of the 10,000 most variable genes after median ratio normalization. (**B**) Sample Spearman correlation. (**C**) Number of up- and down-regulated genes relative to 0 mg L^−1^. DEG thresholds were BH-FDR < 0.05 and |log_2_FC| ≥ 1. (**D**) Central tendencies of mean gene-wise z-scores for each cluster (C1–C6) at 0, 10, 25, and 100 mg L^−1^. (**E**) Heatmap of responsive genes grouped by cluster. Treatment means were log_2_(TPM + 0.5) transformed and gene-wise z-normalized, with colors ranging from blue (−2.0) to red (2.0) representing relative expression levels.

The count matrix contained 52,725 genes and 12 libraries. Sequencing quality and read-mapping statistics for all 12 libraries are summarized in Table S4. After filtering for genes with counts ≥10 in at least 3 samples, 40,374 genes were retained for subsequent analysis. Principal component analysis (PCA) clearly separated the four treatments, with the three replicates within each treatment clustering closely together; PC1 and PC2 explained 28.1% and 24.0% of the variance, respectively (Figure 2A). Sample correlation analysis also indicated that the treatment dose dominated the overall expression structure (Figure 2B).

Differential expression analysis showed that the scale of differential expression expanded with dose. Compared to the control (0 mg L^−1^), 2158 (1367 up-regulated, 791 down-regulated), 2838 (1907 up-regulated, 931 down-regulated), and 3038 (1480 up-regulated, 1558 down-regulated) differentially expressed genes (DEGs) were identified under the 10, 25, and 100 mg L^−1^ Cd treatments, respectively (Figure 2C). The number of repressed genes approached that of induced genes at the high dose, suggesting a shift in the system from active regulation to broad transcriptional constraint.

A total of 5665 genes responded in at least one comparison. These genes were grouped into six dose–response programs (Figure 2D): complex/non-monotonic (C1, 581 genes), monotonic induction (C2, 1125 genes), monotonic repression (C3, 1012 genes), low-dose transient induction (C4, 935 genes), intermediate-dose induction (C5, 1407 genes), and a second complex/non-monotonic group (C6, 605 genes). The transient, intermediate-dose, and non-monotonic groups (C1, C4, C5, and C6) together accounted for 62.3% of responsive genes. This predominance shows that the multi-dose design captured response dynamics that would be missed in a single two-group comparison. The heatmap visualizes the expression patterns of genes in each cluster across the CdCl_2_ gradient (Figure 2E).

### 2.3. Dose-Dependent Pathway Reconfiguration and Metal–Redox Response Networks

**Figure 3:**
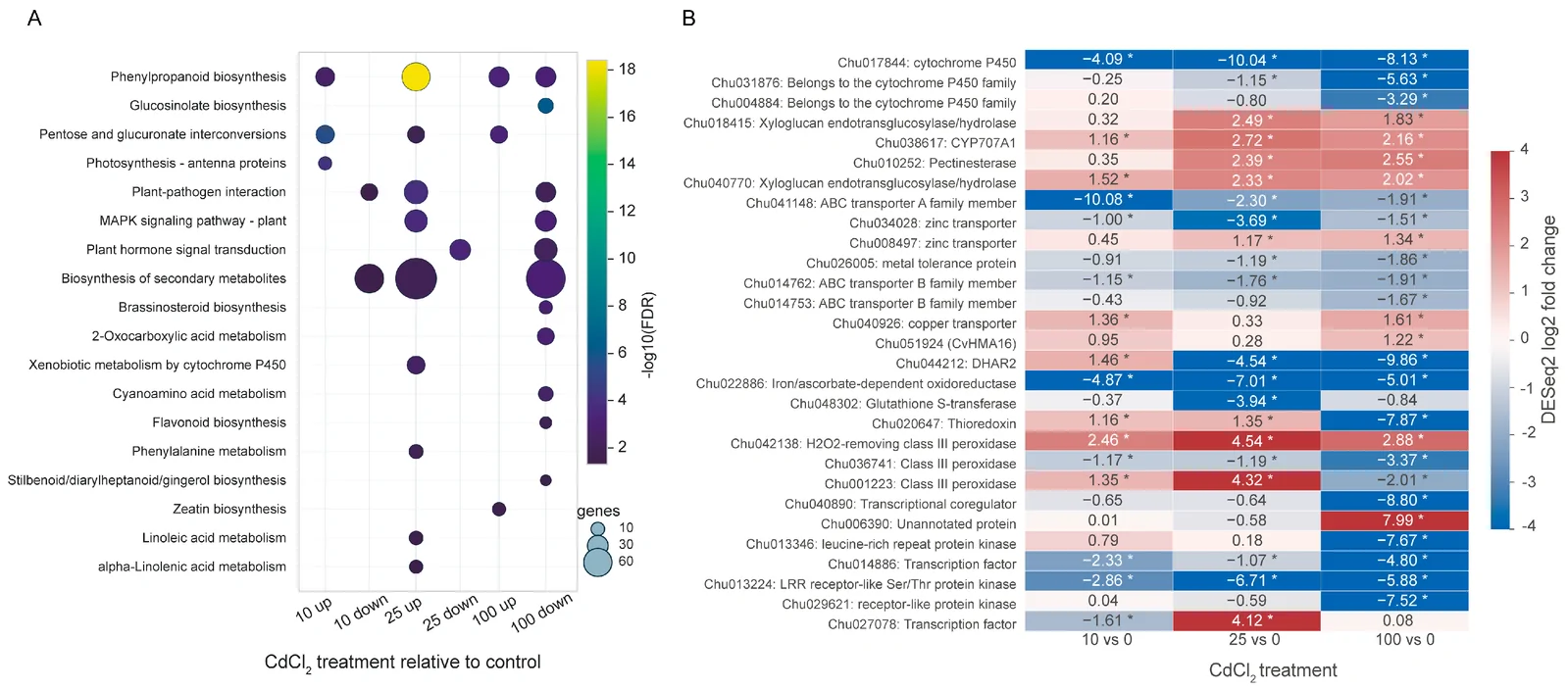
KEGG pathway enrichment and expression profiles of candidate genes under Cd treatment. (**A**) Dot plot of dose- and direction-specific (up or down relative to control) KEGG pathway enrichment. Circle size represents the number of overlapping genes, and the color gradient indicates the significance level as −log_10_(FDR), with yellower colors representing higher significance. (**B**) Heatmap showing log_2_ fold changes of prioritized transport, redox, signaling, and cell-wall candidate genes under 10, 25, and 100 mg L^−1^ CdCl_2_ treatments relative to the control. Red indicates up-regulation, and blue indicates down-regulation. Asterisks indicate comparisons with log_2_FC > 1 or log_2_FC < −1.

Dose- and direction-specific KEGG enrichment revealed marked pathway reconfiguration across the CdCl_2_ gradient (Figure 3A). At 10 mg L^−1^, up-regulated genes were enriched in photosynthesis-antenna proteins, pentose and glucuronate interconversions, phenylpropanoid biosynthesis, and related pathways, consistent with early adjustment of light capture, carbon flux, and cell-wall metabolism. At 25 mg L^−1^, up-regulated genes showed the broadest enrichment of active defense-associated pathways, including phenylpropanoid biosynthesis, plant–pathogen interaction, MAPK signaling, cytochrome P450-mediated xenobiotic metabolism, and linoleic/α-linolenic acid metabolism, whereas plant-hormone signal transduction was enriched among down-regulated genes. At 100 mg L^−1^, down-regulated genes were enriched across glucosinolate biosynthesis, MAPK signaling, brassinosteroid biosynthesis, hormone signaling, plant–pathogen interaction, and several secondary-metabolism pathways; a smaller set of pathways, including phenylpropanoid biosynthesis, pentose and glucuronate interconversions, and zeatin biosynthesis, remained enriched among up-regulated genes. Screening of the DEG union identified 315 candidates annotated for metal transport, detoxification, redox regulation, signaling, or cell-wall remodeling. Their log_2_ fold-change profiles were heterogeneous (Figure 3B): some zinc transporters and peroxidases were induced at particular doses, whereas selected ABC transporters, heavy-metal tolerance proteins, glutathione S-transferases, and thioredoxins were repressed or changed direction across the gradient. These patterns support dose-dependent redistribution of transport and redox programs, but do not establish direct regulatory or transport relationships among the genes.

### 2.4. Transcriptome-Guided Prioritization of CvHMA15 and CvHMA16

**Figure 4:**
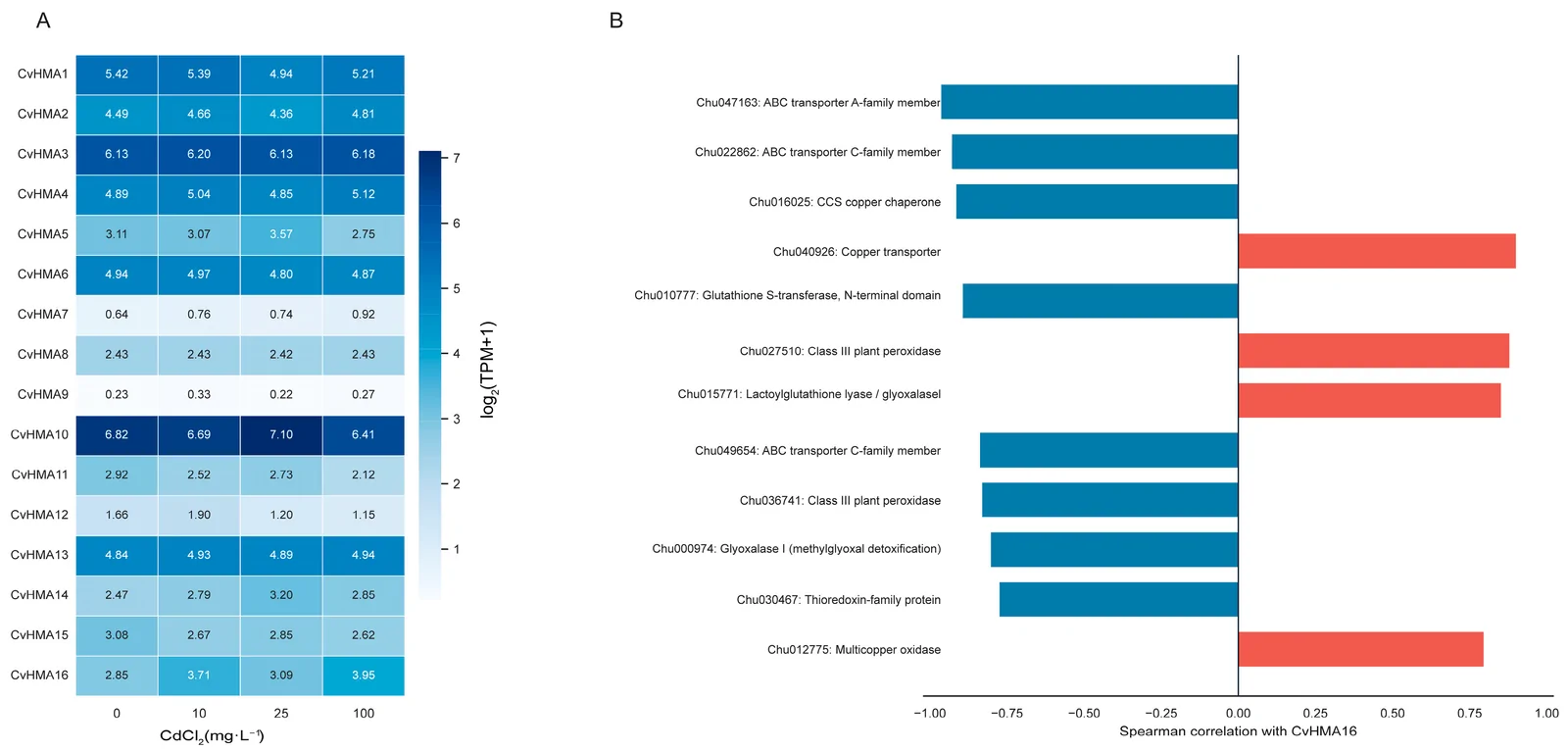
Transcriptome-driven screening of *CvHMA* candidate genes (**A**) Expression heatmap of the 16 *CvHMA* members across four CdCl_2_ dose gradients, highlighting the opposite response patterns of *CvHMA15* and *CvHMA16*; (**B**) Representative transport and redox genes sharing the dose–response profile with *CvHMA16*. With red indicating positive correlations and blue indicating negative correlations.

To examine the contribution of P1B-type ATPase candidates to the Cd response, the dose-resolved transcriptome was cross-referenced with 16 reference-annotated CvHMA sequences. *CvHMA10* had the highest basal abundance but showed relatively small fold changes, whereas *CvHMA14* reached its highest mean abundance at 25 mg L^−1^ (Figure 4A). *CvHMA16* was prioritized because its Cd-responsive RNA-seq profile met the predefined differential-expression criteria at 100 mg L^−1^ CdCl_2_. *CvHMA15* was included as a highly sequence-similar comparator, as its quantified transcript abundance was lower than the control under all three Cd treatments. Sequence comparisons showed that the two candidates encode 766-aa proteins sharing 99.87% amino-acid identity, with a single amino acid difference, whereas their 2301-nt coding sequences differed at four nucleotide positions. To further investigate their genomic relationship, we compared their local genomic contexts on unplaced Scaffold 1458 and Scaffold 1464. Local alignment of these scaffolds, measuring 24,207 bp and 24,138 bp, respectively, yielded 18,697 paired non-gap nucleotide positions with 99.67% identity, covering approximately 77% of each scaffold. High sequence similarity extended beyond the candidate coding regions into neighboring transcripts, including 913 paired positions with complete identity between the downstream transcripts. The log_2_ fold changes in *CvHMA15* at 10, 25, and 100 mg L^−1^ were −0.48, −0.25, and −0.53, respectively (all adjusted *p* < 0.05), but none met the predefined |log_2_FC| ≥ 1 threshold. The corresponding values for *CvHMA16* were 0.95, 0.28, and 1.22; only the 100 mg L^−1^ comparison met both criteria. Mean *CvHMA16* abundance increased from 6.24 TPM in the control to 12.07 and 14.45 TPM at 10 and 100 mg L^−1^, whereas *CvHMA15* decreased from 7.45 TPM to 5.37 and 5.14 TPM. qRT-PCR analysis confirmed the non-monotonic Cd-responsive expression pattern of *CvHMA16*, with significantly higher transcript levels at 10 and 100 mg L^−1^ than at 0 and 25 mg L^−1^ CdCl_2_ (*p* < 0.05), closely consistent with the RNA-seq profile (Figure S1). Across the 12 libraries, *CvHMA16* showed significant correlations with selected copper-transport, ABC-transporter, peroxidase, thioredoxin, and glyoxalase genes (Figure 4B). These correlations describe shared dose-structured profiles rather than direct regulation. The combination of high protein similarity and divergent Cd-responsive transcription motivated the subsequent evolutionary analysis and yeast comparison of *CvHMA15* and *CvHMA16*.

### 2.5. Genome-Wide Identification, Evolution, and Structural Characterization of the CvHMA Family

**Figure 5:**
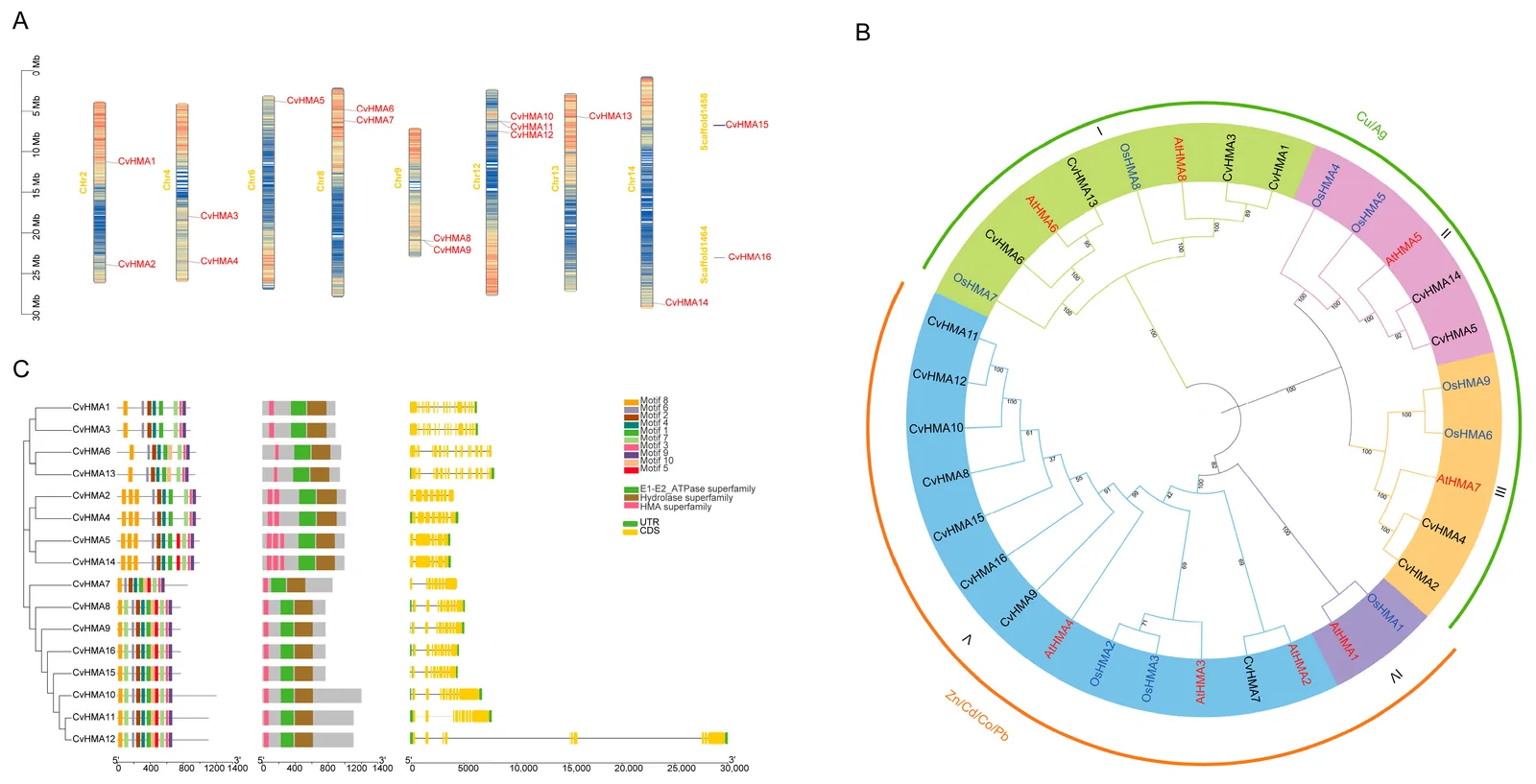
Genomic distribution, evolutionary relationship, and structural characterization of the *CvHMA* gene family. (**A**) Chromosomal distribution of *CvHMA* genes. Chromosome numbers are shown at the left of each bar, with the scale indicated in megabases (Mb). (**B**) Phylogenetic tree of HMA members. The tree is divided into five clades. Clades I, II, and III belong to the Cu/Ag subgroup (indicated by green brackets), whereas clades IV and V belong to the Zn/Cd/Co/Pb subgroup (indicated by yellow brackets). *OsHMAs* are shown in blue font, and *AtHMAs* are shown in red font. Bootstrap support was assessed using 1000 replicates. (**C**) Classification and structural analysis of the *CvHMA* gene family. From left to right: a neighbor-joining phylogenetic tree of *CvHMAs* (different colors represent different subgroups); conserved motifs of CvHMA proteins (the 10 predicted motifs are represented by boxes in different colors); distinct conserved domains of *CvHMAs* (indicated by differently colored boxes); and exon-intron structures of *CvHMA* genes (gray lines indicate introns). Sequence logos of the 10 conserved motifs are provided in Figure S3.

Genome-wide analysis identified 16 CvHMA loci in *Cardamine violifolia*. The predicted proteins ranged from 765 to 1201 amino acids and had theoretical pI values of 5.15–9.05; DeepLoc 2.0 predicted several localization categories, including plastid, lysosome/vacuole, endoplasmic reticulum, cell membrane, and mitochondrial/plastid locations (Table S1). Phylogenetic analysis divided the proteins into five clades (Figure 5B). Groups I–III contained the Cu/Ag-associated members, whereas Group V contained eight Zn/Cd/Co/Pb-associated CvHMAs (*CvHMA7*–*CvHMA12*, *CvHMA15*, and *CvHMA16*), compared with three Arabidopsis members in the corresponding branch. No CvHMA was placed with the AtHMA1-containing Group IV. Chromosomal mapping located *CvHMA* genes on chromosomes 2, 4, 6, 8, 9, 12, 13, and 14, with *CvHMA15* and *CvHMA16* retained on unplaced scaffolds (Figure 5A). Several genes were physically clustered, but proximity alone was not treated as proof of tandem duplication. Protein architecture was conserved: all members contained the expected E1–E2 ATPase and hydrolase domains and shared Motifs 1, 2, 6, and 9, whereas exon–intron organization varied among genes (Figure 5C).

Comparative genomic analysis recovered HMA-associated collinear anchors between *Cardamine* and *Arabidopsis thaliana*, including *CvHMA2*/*CvHMA4* with *AtHMA7*, *CvHMA5*/*CvHMA14* with *AtHMA5*, and *CvHMA6*/*CvHMA13* with *AtHMA6* (Figure S2B). These anchors indicate conserved genomic relationships rather than establishing one-to-one orthology. Four chromosome-anchored *CvHMA* collinear pairs—*CvHMA2*/*CvHMA4*, *CvHMA1*/*CvHMA3*, *CvHMA5*/*CvHMA14*, and *CvHMA6*/*CvHMA13*—had NG86 dN/dS estimates ranging from 0.071 to 0.160, consistent with purifying selection on their coding sequences (Figure S2A; Table S3).

### 2.6. Tissue Expression Profiles of CvHMA Genes

**Figure 6:**
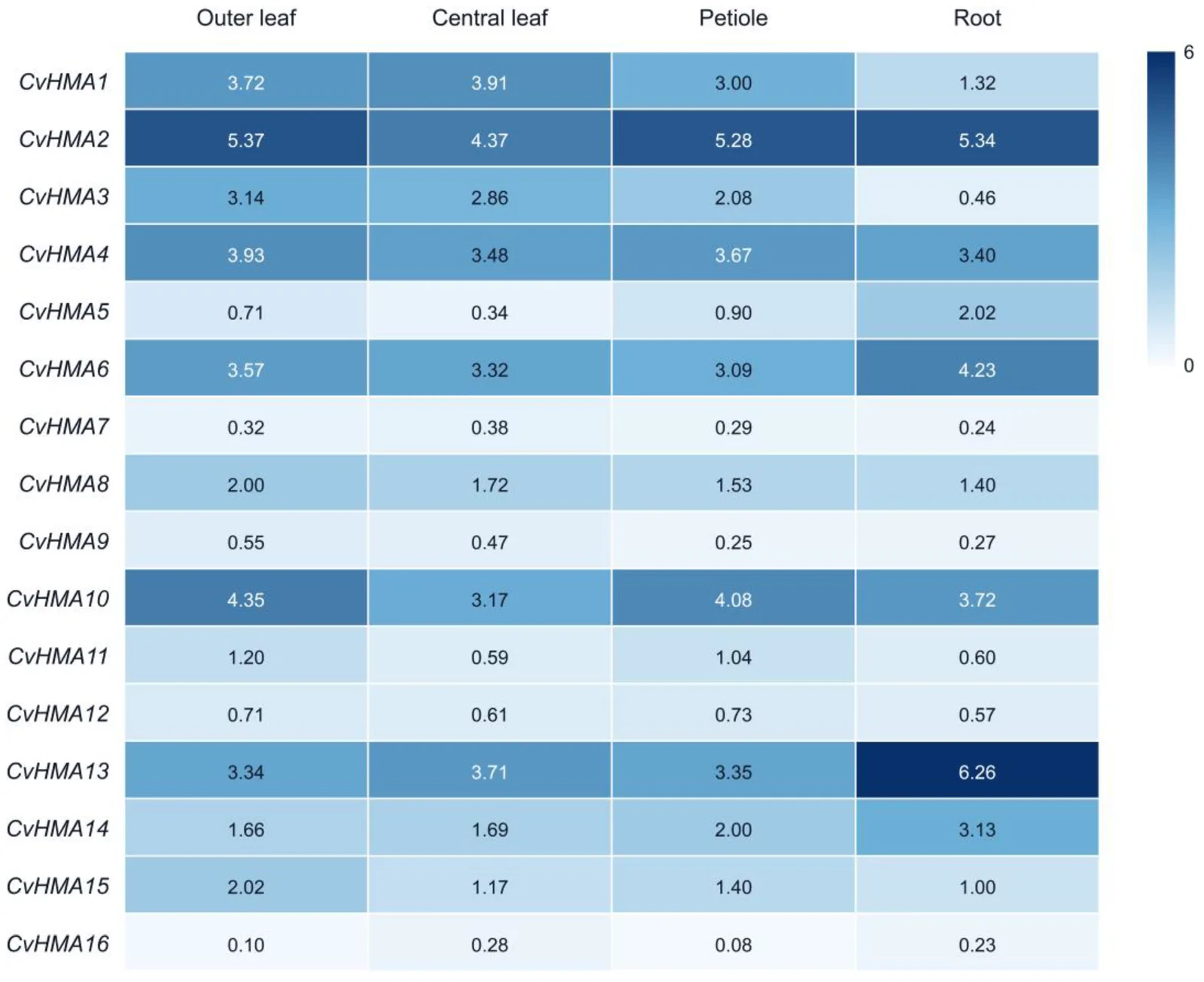
Absolute tissue-expression profiles of *CvHMA* genes in *Cardamine violifolia*. The heatmap shows absolute expression of the 16 *CvHMA* genes in outer leaves, central leaves, petioles, and roots. Values and cell colors represent log_2_(TPM + 1), with darker blue indicating higher transcript abundance.

TPM-based transcript abundance revealed distinct but overlapping tissue-expression patterns among *CvHMA* genes (Figure 6). *CvHMA13* showed the highest root abundance, and *CvHMA6* and *CvHMA14* were also relatively enriched in roots. *CvHMA2* was abundant in outer leaves, petioles, and roots, whereas *CvHMA10* was abundant in outer leaves and petioles. *CvHMA1* and *CvHMA3* were more abundant in leaves than in roots. These profiles indicate tissue preferences but do not establish transport direction or functional specialization.

*CvHMA16* had low TPM-based abundance in all four tissues despite its Cd inducibility in the treatment experiment, whereas *CvHMA15* showed modestly higher abundance in outer leaves than in the other tissues. Because the tissue atlas and Cd-gradient experiment were generated independently, the datasets were used as complementary descriptive evidence and were not combined to infer a spatiotemporal transport pathway.

### 2.7. Yeast Functional Verification of CvHMA15 and CvHMA16

**Figure 7:**
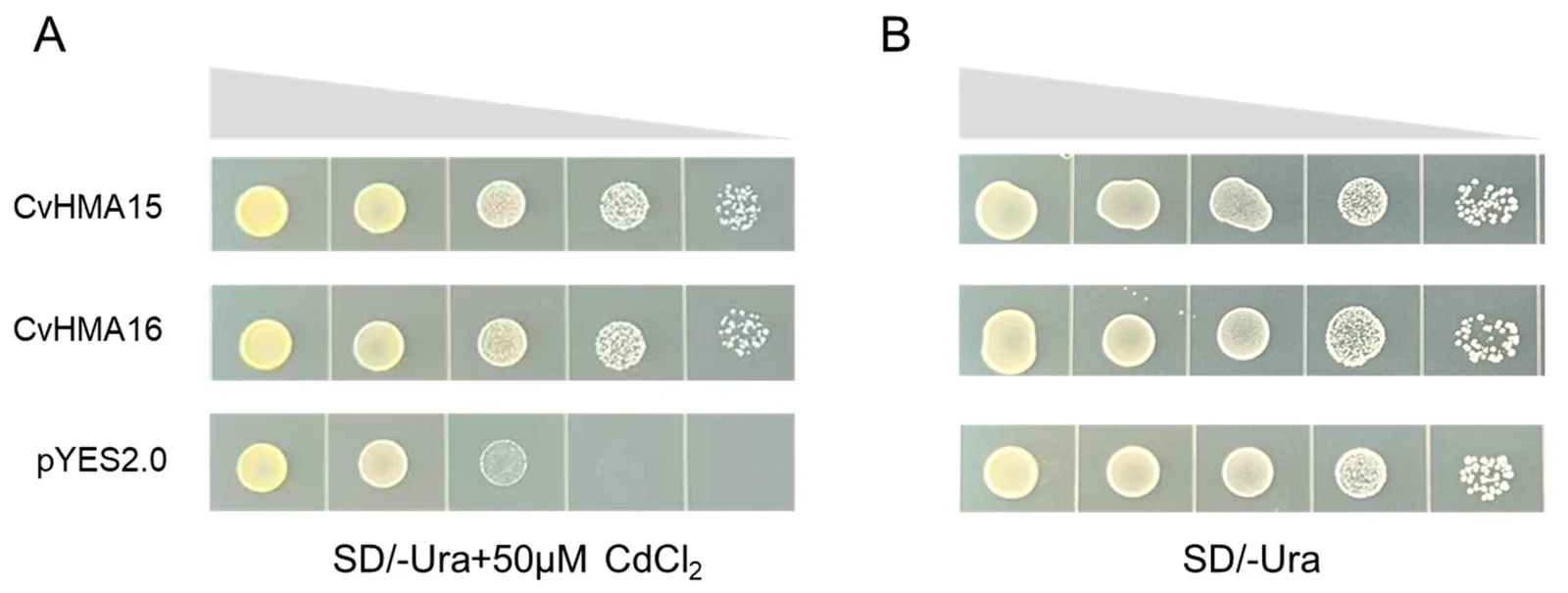
Functional verification of heavy metal tolerance of *CvHMA15* and *CvHMA16* in yeast. (**A**) Yeast cells transformed with *CvHMA15*, *CvHMA16*, and the empty pYES2.0 vector (as a control) were subjected to five serial dilutions (indicated by the grey wedges at the top) and spotted onto SD/-Ura medium supplemented with 50 μM CdCl_2_. (**B**) the control SD/-Ura medium without CdCl_2_ to observe and compare their growth phenotypes.

On Cd-free selective medium containing 2% galactose, yeast carrying pYES2.0-CvHMA15, pYES2.0-CvHMA16, or the empty pYES2.0 vector showed visually similar growth across the dilution series (Figure 7B). In the presence of 50 μM CdCl_2_, empty-vector yeast showed strongly reduced growth, particularly at higher dilutions, whereas yeast expressing either *CvHMA15* or *CvHMA16* retained visible colony growth across the tested cell densities (Figure 7A).

## 3. Discussion

### 3.1. Dose Dependent Root and Transcriptome Responses to Cadmium

The CdCl_2_ gradient produced a biphasic root-length response in *Cardamine violifolia*. Exposure to 10 mg L^−1^ promoted root elongation, whereas 100 mg L^−1^ reduced root length and surface area relative to the low-dose treatment. Similar low-dose stimulation followed by inhibition at higher exposure has been described as a hormetic response to Cd [23]. Nevertheless, the present phenotypes do not directly establish the physiological mechanisms underlying this response. The filter-paper system also lacks soil-like buffering and may be affected by localized exposure or drying. The selected concentrations should therefore be interpreted as an experimental stress gradient rather than a direct representation of field exposure.

The transcriptome analysis resolved the molecular programs underlying these dose-dependent phenotypes. The number of DEGs increased across the Cd gradient, but the functional composition of the response changed with dose. At 10 mg L^−1^, transcriptional adjustment involved photosynthetic, carbon-metabolic, phenylpropanoid, and cell-wall-associated processes. The 25 mg L^−1^ treatment produced the broadest activation of defense-related pathways, including phenylpropanoid biosynthesis, MAPK signaling, plant–pathogen interaction, lipid signaling, and xenobiotic metabolism. At 100 mg L^−1^, the increasing contribution of repressed genes and suppression of multiple signaling and secondary-metabolism pathways were consistent with the observed growth inhibition. The six expression trajectories further showed that 62.3% of responsive genes followed transient, intermediate-dose, or non-monotonic patterns, demonstrating that the response cannot be represented by a simple linear increase in stress severity. Coordinated regulation of sulfur metabolism, redox processes, signaling, transport, phenylpropanoid metabolism, and cell-wall remodeling has also been reported in Arabidopsis and Tartary buckwheat under Cd stress [24,25]. These results place metal transport within a broader acclimation network and provide the molecular rationale for using the transcriptome to prioritize responsive HMA genes.

### 3.2. Transcriptome Guided Prioritization Reveals Regulatory Divergence Within a Conserved HMA Pair

Transcriptome-guided prioritization distinguished basal transcript abundance from Cd responsiveness. Although *CvHMA10* had the highest control abundance, *CvHMA16* showed a larger Cd-associated response and met the combined FDR and effect-size criteria at 100 mg L^−1^. *CvHMA15* provided a highly sequence-similar comparison despite its smaller changes below the predefined effect-size threshold. The extensive similarity of their coding sequences and scaffold neighborhoods raises the possibility of an allelic relationship, but does not resolve their locus identity. Moreover, separately quantified RNA-seq signals from highly similar sequences require caution because ambiguous read assignment has not been excluded. The observed profiles therefore motivate sequence-discriminating expression analyses rather than establish allele-specific regulation. Studies of HMA4 in *Arabidopsis halleri* and *Noccaea caerulescens* demonstrate that copy-number and cis-regulatory changes can contribute to metal adaptation [15,16,17], but these precedents do not establish the mechanism operating in the present candidates.

Phylogenetic placement and conserved protein architecture provide a biological basis for investigating the involvement of *CvHMA15* and *CvHMA16* in Cd responses. The Zn/Cd/Co/Pb-associated HMA clade includes HMA3 proteins involved in vacuolar Cd sequestration [12,13] and *HMA2*/*HMA4* proteins associated with metal efflux and long-distance transport [26,27,28], highlighting the diverse contributions of HMA transporters to metal homeostasis. Within this context, the low expression of *CvHMA16* in the independent tissue atlas and its increased expression under Cd treatment in the seedling experiment suggest a potentially condition-dependent contribution to Cd responses. Predicted differences in upstream cis-regulatory motifs provide additional hypotheses for investigating the regulatory patterns associated with the two candidates. Together, these findings connect transcriptome-guided candidate identification with functional evidence from yeast and identify *CvHMA15* and *CvHMA16* as candidates for further investigation of Cd homeostasis in *C. violifolia*. Establishing their subcellular localization and physiological contributions in plants will help clarify how these activities relate to Cd transport, tolerance, and accumulation.

### 3.3. CvHMA15 and CvHMA16 Show Cadmium Tolerance Related Activity in Yeast

Heterologous expression of either *CvHMA15* or *CvHMA16* improved yeast growth under 50 μM CdCl_2_ relative to the empty-vector control, whereas growth was visually similar among transformants on Cd-free medium (Figure 7). Both constructs therefore showed Cd-tolerance-related activity in yeast despite the single amino acid difference between their encoded proteins. However, this qualitative phenotype does not establish equivalent intrinsic protein activity or determine whether their contrasting plant transcript profiles are associated with functional differentiation in planta. The combined expression and yeast results provide a basis for investigating the regulation and physiological contributions of these two highly similar candidates in the native plant.

The yeast spot assay (Figure 7) provides direct evidence of Cd-tolerance-related activity but cannot delineate the corresponding mechanism in planta. Within the Zn/Cd/Co/Pb-associated HMA clade, *AtHMA3* and *OsHMA3* primarily mediate vacuolar Cd sequestration [12,29,30], whereas HMA2/HMA4 proteins participate in plasma-membrane efflux and long-distance metal transport [26,27,28]. The similar yeast phenotypes of *CvHMA15* and *CvHMA16* are therefore compatible with retained transporter activity, but they do not distinguish Cd uptake restriction, cellular efflux, intracellular sequestration, or differences in substrate specificity and affinity. Future studies combining CRISPR/Cas9-mediated targeted mutagenesis, native-plant overexpression, subcellular localization, Cd-accumulation measurements, and direct transport assays will be required to determine their membrane localization, transport direction, biochemical properties, and contributions to systemic Cd accumulation in *C. violifolia*.

## 4. Materials and Methods

### 4.1. Plant Materials, Cd Treatments, and Tissue Collection

*Cardamine violifolia* seeds were obtained from the Wuhan Agricultural Science Research Institute. Seeds were naturally air-dried and stored in an ultra-dry seed cabinet (iHZDs-1600II, Beijing Biofuture Bio-Tech Co., Ltd., Beijing, China) at approximately 25 °C and 1–5% relative humidity. Anhydrous CdCl_2_ (Shanghai Aladdin Biochemical Technology Co., Ltd., Shanghai, China) was dissolved in distilled water to prepare 0, 10, 25, and 100 mg L^−1^ treatment solutions, with the highest concentration designed to induce acute stress and capture the full transcriptional response trajectory of this metal-tolerant species; distilled water served as the control.

For each treatment, seeds were soaked in 5 mL of the corresponding solution for 24 h in a constant-temperature light incubator. They were then placed on filter paper moistened with the same solution and maintained at 25/20 °C (day/night), a 16 h light/8 h dark photoperiod, and 60% relative humidity. The corresponding treatment solution was added daily to maintain moisture. After 7 d, root systems were scanned and analyzed, after which whole seedlings were collected, frozen in liquid nitrogen, and stored at −80 °C. Each treatment comprised three independent biological replicates, with eight seedlings measured per replicate.

### 4.2. Transcriptome Sequencing and Differential Expression Analysis

Total RNA was extracted from seedlings exposed to 0, 10, 25, and 100 mg L^−1^ CdCl_2_. Three independent biological replicates were sequenced per treatment, yielding 12 libraries. Library preparation and sequencing were performed by Beijing Biomarker Technologies Co., Ltd. (Beijing, China). After quality filtering, clean reads were mapped to the reference genome with STAR v2.7 [31], and gene-level counts were generated with featureCounts [32]. Genes with at least 10 counts in three or more libraries were retained, leaving 40,374 of 52,725 genes. Differential expression was estimated with the DESeq2 framework as implemented in PyDESeq2 [33,34], using treatment as the design factor and 0 mg L^−1^ as the reference. Genes with a Benjamini–Hochberg FDR < 0.05 and |log_2_ FC| ≥ 1 were classified as DEGs. TPM values were used for abundance visualization. PCA was performed on the 10,000 most variable genes after median-ratio normalization. For trajectory analysis, treatment means were log_2_(TPM + 0.5) transformed, standardized gene-wise, and partitioned into six clusters by k-means clustering (random seed 260724; 50 initializations). KEGG enrichment was tested separately for up- and down-regulated genes using a one-sided Fisher exact test against the expressed, annotated background, followed by BH correction. Spearman correlations with *CvHMA16* were calculated across the 12 log_2_-transformed normalized-count profiles; associations with FDR < 0.05 and |ρ| ≥ 0.75 were retained as descriptive co-expression candidates.

### 4.3. qRT-PCR Validation

To assess the expression trends observed in the RNA-seq analysis, total RNA was extracted from *Cardamine violifolia* seedlings exposed to 0, 10, 25, and 100 mg L^−1^ CdCl_2_ using the HiPure Universal RNA Mini Kit (Magen, Guangzhou, China). First-strand cDNA was synthesized using HiScript III RT SuperMix for qPCR (Vazyme Biotech, Nanjing, China). The resulting cDNA was diluted fivefold and used as the template for qRT-PCR with ChamQ Universal SYBR qPCR Master Mix (Vazyme Biotech, Nanjing, China). The amplification program consisted of an initial denaturation at 95 °C for 5 min, followed by 40 cycles of 95 °C for 10 s and 60 °C for 20 s. A melting-curve analysis was performed after amplification to assess amplification specificity. *CvHMA4*, *CvHMA10*, and *CvHMA16* were selected for analysis. Relative expression levels were calculated using the 2^−ΔΔCt^ method, with Cvactin as the internal reference gene. The expression stability of the reference gene Cvactin was further confirmed by our RNA-seq data, which showed minimal fluctuation across the 0–100 mg L^−1^ CdCl_2_ gradient (all log_2_|FC| < 0.3). Three biological replicates were analyzed per treatment. Primer sequences are provided in Table S2.

### 4.4. Genome-Wide Identification of CvHMA Genes

Protein sequences and gene annotations were obtained from the chromosome-level *Cardamine enshiensis* reference assembly GWHBHEU00000000, originally deposited in the National Genomics Data Center under project PRJCA002827 [22]. For consistency with the experimental material, candidate genes are designated *CvHMA*. *Arabidopsis thaliana* HMA proteins were used as BLASTP queries (E-value ≤ 1 × 10^−10^) [35]. Candidate sequences were screened using the PF00403 HMA-domain, PF00122 E1–E2 ATPase-domain, and PF00702 hydrolase-domain profiles from Pfam [36] and further verified with NCBI CDD [37]. Protein length, molecular weight, theoretical pI, instability index, aliphatic index, and GRAVY were calculated in TBtools v 2.466 [38]. Subcellular localization was predicted with Deep Loc 2.0 [39,40] and treated as computational evidence requiring experimental validation.

### 4.5. Phylogenetic and Sequence Identity Analysis

Full-length HMA protein sequences from *Arabidopsis thaliana* and *Oryza sativa* were obtained from TAIR and Phytozome, respectively [41,42]. Sequences were aligned in MEGA X v10.1.7, and a neighbor-joining tree was inferred with 1000 bootstrap replicates [43]. The tree was visualized in iTOL v7 [44]. Pairwise protein identity was calculated from aligned non-gap positions; the *CvHMA15*/*CvHMA16* comparison contained 766 aligned positions.

### 4.6. Conserved Motifs, Domains, Promoters, and Gene Structure

Conserved domains were identified with NCBI Batch CD-Search [45]. Conserved motifs were predicted with MEME Suite v5.5.9 using default parameters and a maximum of 10 motifs [46]. Genomic coordinates, exon–intron structures, and strand orientations were extracted from the GFF3 annotation. Promoter sequences were defined as the 2000 bp immediately upstream of each annotated start site and analyzed with PlantCARE (http://bioinformatics.psb.ugent.be/webtools/plantcare/html/, accessed on 15 October 2026) [47]. Predicted elements were grouped by reported stress- and hormone-associated categories and visualized in TBtools. These annotations were used to generate regulatory hypotheses and were not treated as direct evidence of promoter activity.

### 4.7. Tissue Expression Analysis

RNA-seq datasets for outer leaves, central leaves, petioles, and roots were obtained from NGDC project PRJCA019808. Clean reads were mapped to the same reference assembly with STAR [31], and gene counts were generated with featureCounts v2.1.1 [32]. Transcript abundance was expressed as TPM. CvHMA profiles were visualized as absolute log_2_(TPM + 1) values so that color differences retained information about both tissue preference and abundance. Because this public tissue atlas was independent of the Cd-gradient experiment, it was analyzed descriptively and was not used to infer tissue-specific Cd responses.

### 4.8. Yeast Expression and Cd-Tolerance Assay

The full-length coding sequences of *CvHMA15* and *CvHMA16* were amplified from cDNA synthesized from whole-seedling RNA using Phanta Max Super-Fidelity DNA Polymerase (Vazyme). The sequence accuracy of the amplified products was verified by Sanger sequencing before they were separately cloned into the pYES2.0 yeast expression vector using an In-Fusion cloning strategy. The recombinant pYES2.0-CvHMA15 and pYES2.0-CvHMA16 plasmids, together with the empty pYES2.0 vector, were separately transformed into strain BY4741 using the lithium acetate method. Transformants were selected on SD/-Ura solid medium and incubated at 28 °C for 3–5 d. To ensure the consistency and reproducibility of the observed Cd-tolerance phenotype, three independent transformant colonies for each construct and the empty vector control were randomly picked and tested. Fresh single colonies, approximately 2–3 mm in diameter, were picked and resuspended in 1 mL of sterile 0.9% (*w*/*v*) NaCl solution. The cell suspensions were adjusted to an OD_600_ of 1.0 and serially diluted 10-fold to obtain five cell densities (10^0^–10^−4^). A 10 μL aliquot of each dilution was spotted onto selective plates containing 2% galactose, with or without 50 μM CdCl_2_ (a concentration determined in preliminary tests to be strongly inhibitory to the empty-vector control). The plates were incubated at 28 °C for 3 day before imaging. Yeast transformed with the empty pYES2.0 vector was used as the negative control.

### 4.9. Statistical Analysis

Root morphology was measured for 24 seedlings per treatment, arranged as three independent biological replicates of eight seedlings. The mean of the eight seedlings within each replicate was treated as the experimental unit (*n* = 3). Root systems were scanned and quantified with the Keheng Root Analysis System (Keheng, Shenzhen, China). Data are presented as mean ± SD of biological-replicate means. qRT-PCR relative-expression values were log_2_ transformed and analyzed using the same Welch ANOVA. Figures were assembled in GraphPad Prism 9.0 and vector-graphics software.

## 5. Conclusions

Dose-resolved phenotyping and transcriptomics showed that *Cardamine violifolia* seedlings exhibit a biphasic root response and extensive, non-linear transcriptional remodeling under Cd exposure. Integrating these responses with reference-annotated HMA sequences prioritized *CvHMA15* and *CvHMA16* as highly sequence-similar candidates with contrasting quantified transcript profiles under Cd treatment. Heterologous expression of either candidate improved yeast growth under 50 μM CdCl_2_, supporting Cd-tolerance-related activity in this experimental system. Their genomic relationship, regulatory differentiation, transport properties, and contributions to Cd tolerance and accumulation in planta remain to be established.

## Data Availability

The raw sequence data reported in this paper have been deposited in the Genome Sequence Archive (Genomics, Proteomics & Bioinformatics 2025) in National Genomics Data Center (Nucleic Acids Res 2025), China National Center for Bioinformation/Beijing Institute of Genomics, Chinese Academy of Sciences (GSA: CRA035742) that are publicly accessible at https://ngdc.cncb.ac.cn/gsa (accessed on 15 October 2026).

## Supplementary Materials

The following supporting information can be downloaded at: https://www.mdpi.com/article/10.3390/plants15213251/s1. Figure S1. qRT-PCR validation of the RNA-seq data, Figure S2. Evolutionary and comparative genomics analysis of the CvHMA family, Figure S3. Sequence logos of 10 conserved motifs in CvHMA proteins identified by MEME, Figure S4. Quantitative profiling of environmental adaptation-related cis-acting regulatory elements in the promoters of *CvHMA* genes, Table S1 Physicochemical properties and subcellular localization of the 16 CvHMAs identified in *Cardamine violifolia*, Table S2. Sequences of primers used for qRT-PCR validation in this study, Table S3. Collinearity and recalculated selection ratios, Table S4. Summary of RNA-sequencing data quality and read mapping statistics.

## Abbreviations

The following abbreviations are used in this manuscript:

HMA	heavy metal ATPases
HMM	Hidden Markov modeling
ROS	reactive oxygen species
MW	molecular weight
pI	isoelectric point
NJ	Neighbor-Joining
