# Supplementary material for: Cadmium Dose-Responsive Transcriptomics Prioritizes CvHMA15 and CvHMA16 in Cardamine violifolia
Source: Plants (Basel). 2026 Oct 22;15(21):3251. doi: 10.3390/plants15213251 (PMC13765353; doi:10.3390/plants15213251)
Supplement: Supplementary file 1 [file plants-15-03251-s001.zip › plants-4601750-supplementary.pdf]

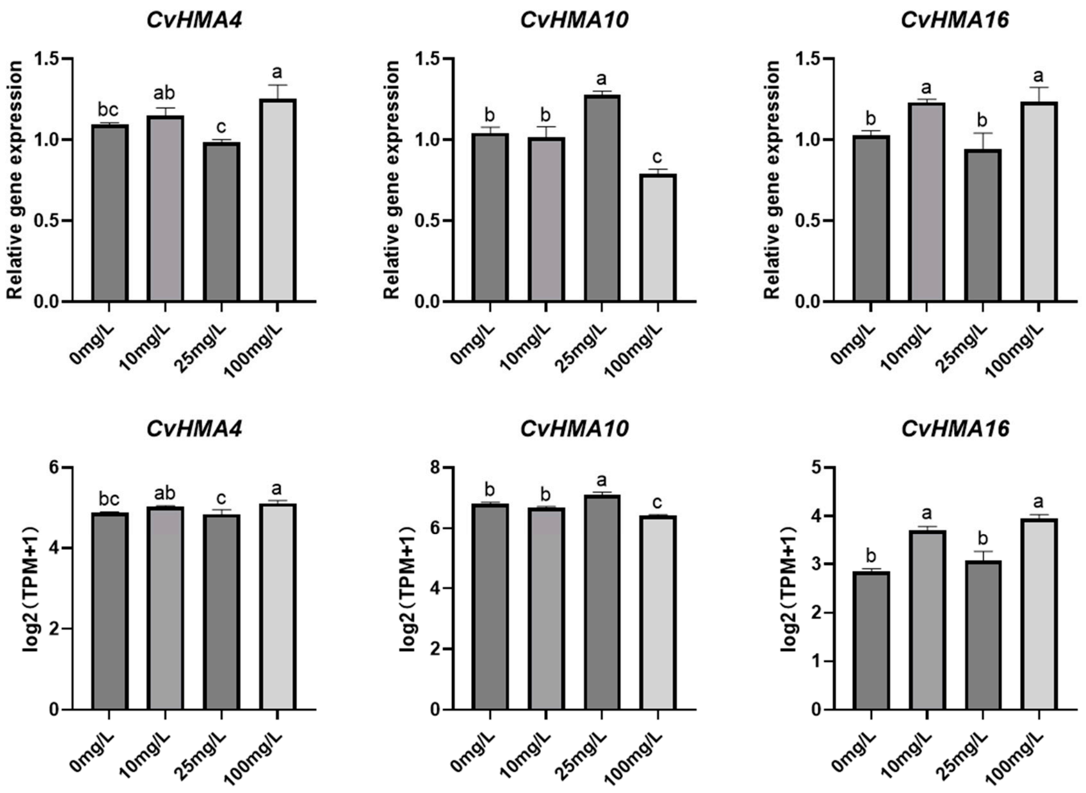

Figure S1. qRT-PCR validation of the RNA-seq data. The upper panel shows the relative gene expression of *CvHMA4*, *CvHMA10*, and *CvHMA16* analyzed by qRT-PCR. The lower panel displays the corresponding transcript abundance represented by log2(TPM+1) from the RNA-seq data. Data are shown as the mean ± SD of three biological replicates.

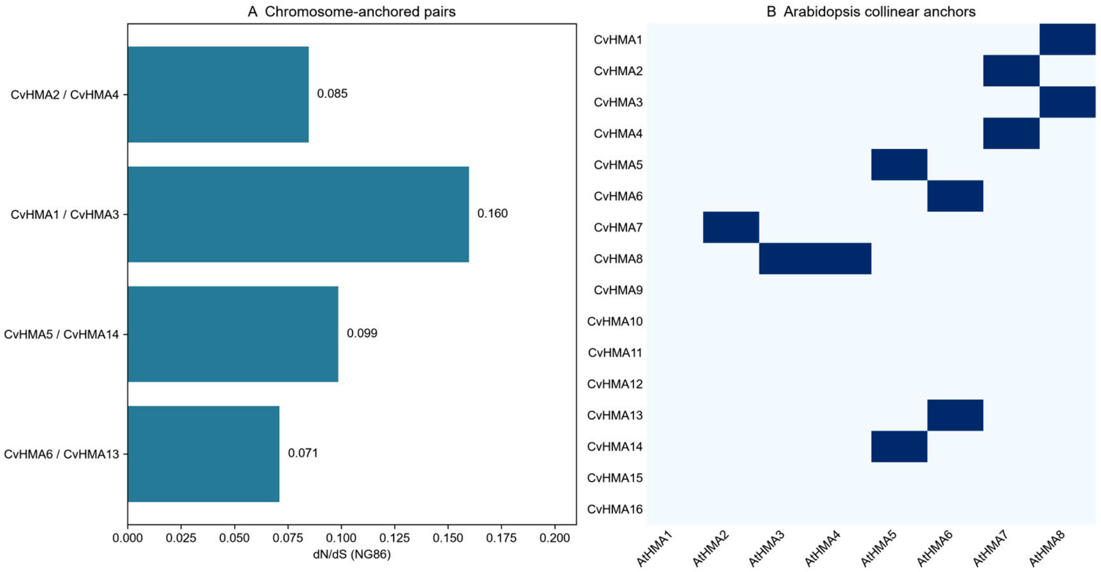

Figure S2. Evolutionary and comparative genomics analysis of the CvHMA family. (A) Recalculated NG86 dN/dS ratios for four chromosome-anchored HMA collinear pairs. (B) HMA-associated Arabidopsis anchors from available MCScanX blocks. Filled cells indicate a recovered anchor, not a one-to-one orthology test. *CvHMA2*/*CvHMA3* names have been reconciled to submitted Table S1 using stable gene IDs. Estimates are computational sequence comparisons, not replicated experimental measurements.

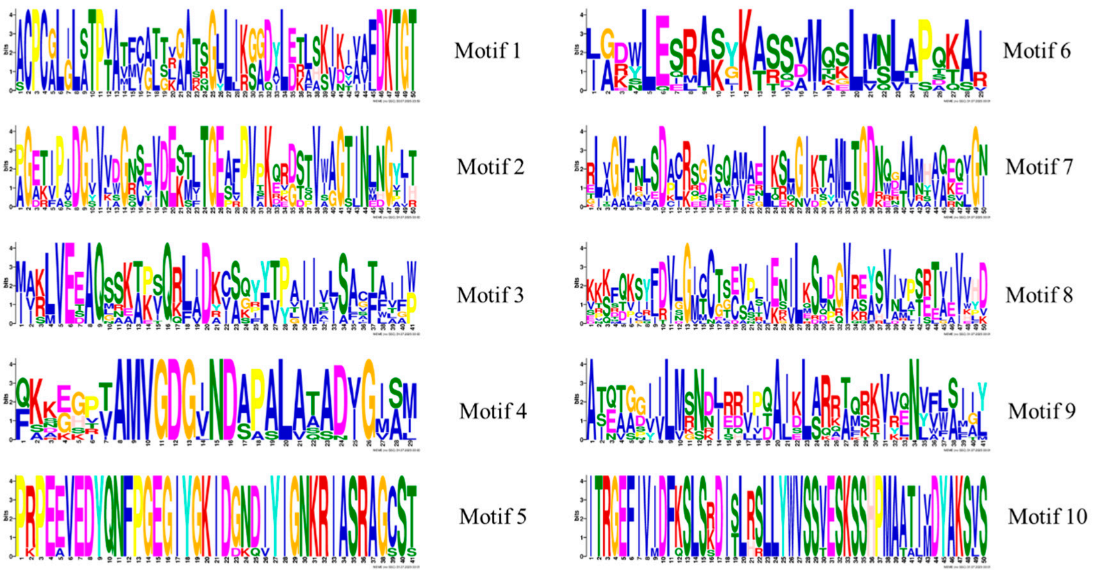

Figure S3. Sequence logos of 10 conserved motifs in CvHMA proteins identified by MEME.

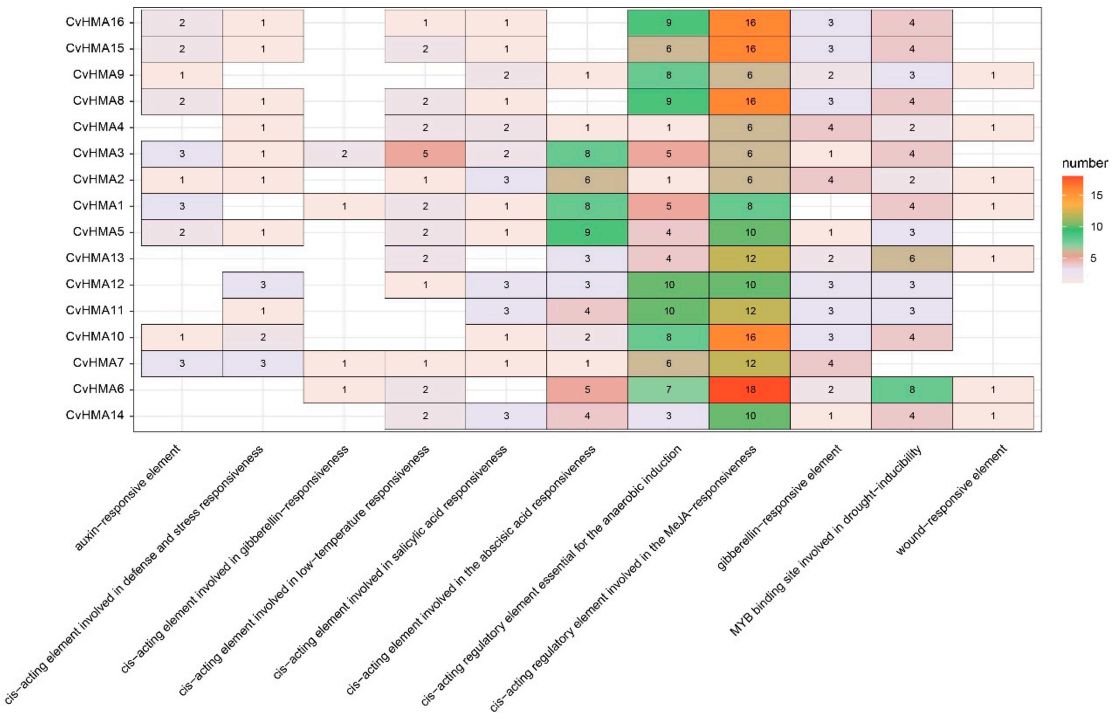

Figure S4. Quantitative profiling of environmental adaptation-related cis-acting regulatory elements in the promoters of CvHMA genes.

Table S1 Physicochemical properties and subcellular localization of the 16 CvHMAs identified in *Cardamine violifolia*.

Name	Chr.	Genomic location	Protein length	M.W (kDa)	pI	Instability Index	Aliphatic Index	GRAVY	Subcellular localization
CvHMA1	2	7409003...7415285	885	94.23	5.85	39.74	105.1	0.164	Plastid
CvHMA2	2	20078246...20082352	1013	108.42	5.15	32.35	103.05	0.222	Lysosome/Vacuole
CvHMA3	4	13905179...13911552	886	94.39	5.75	37.73	103.65	0.169	Plastid
CvHMA4	4	19516120...19520652	1012	108.17	5.15	31.94	104.42	0.248	Lysosome/Vacuole
CvHMA5	6	659141...662927	995	108.33	6.05	32.71	104.07	0.166	Plastid
CvHMA6	8	2614169...2621820	954	100.4	8.97	37.44	95.46	0.082	Plastid
CvHMA7	8	4040508...4044911	850	91.34	7.38	33.5	87.55	-0.127	Cell membrane
CvHMA8	9	13799652...13804803	765	82.8	8.17	31.74	109.36	0.215	Lysosome/Vacuole
CvHMA9	9	13812326...13817419	765	82.77	8.17	31.74	109.49	0.219	Lysosome/Vacuole
CvHMA10	12	3931323...3938092	1201	130.44	6.15	36.13	89.27	-0.143	Endoplasmic reticulum
CvHMA11	12	4022819...4030499	1106	119.9	6.48	38.48	90.07	-0.129	Endoplasmic reticulum
CvHMA12	12	5171507...5201382	1105	119.79	6.48	38.8	90.15	-0.126	Endoplasmic reticulum
CvHMA13	13	2874067...2881979	940	99.1	9.05	35.73	96.46	0.079	Mitochondrion|Plastid
CvHMA14	14	27883789...27887620	995	108.35	6.32	32.72	104.66	0.172	Plastid
CvHMA15	Scaffold 1458	14131...18606	766	82.9	8.3	31.71	109.35	0.213	Lysosome/Vacuole
CvHMA16	Scaffold 1464	9863...14449	766	82.88	8.17	30.89	109.35	0.215	Lysosome/Vacuole

Table S2. Sequences of primers used for qRT-PCR validation in this study

Primer Name	Sequence (5'–3')
CvHMA4-F	CGGCAAGGGCAGTCGCTAA
CvHMA4-R	TAGAGCAGGAGAGTCATTAA
CvHMA10-F	GGGGACAGTGAAACAAAGC
CvHMA10-R	AATCCGAACTATCAGACTCTACAT
CvHMA16-F	GTCACTCTCTAGAGATATAAGCCTACA
CvHMA16-R	ATAGATATCGTTCCCATCAATCTTCCCG
Cvactin-F	TTTGGAGGCTCTGTGCTTTC
Cvactin-R	TGTACGGCATATGCTTGCTC

Note: Cvactin was used as the internal reference gene for expression normalization.

Table S3 Collinearity and recalculated selection ratios

Pair	Codons	dN	dS	dN/dS
CvHMA6/CvHMA13	938	0.005196	0.073062	0.0711
CvHMA5/CvHMA14	995	0.011225	0.113760	0.0987
CvHMA1/CvHMA3	885	0.015129	0.094691	0.1598
CvHMA2/CvHMA4	1012	0.010659	0.125795	0.0847
CvHMA15/CvHMA16	766	0.000566	0.005676	0.0997

Table S4. Summary of RNA-sequencing data quality and read mapping statistics.

Sample	total Reads	Q20(%)	Q30(%)	GC(%)	Mapped_Reads(%)
0mg/L-1	39,342,342	99.49	97.87	44.03	92.09
0mg/L-2	35,243,327	99.60	98.30	43.99	92.29
0mg/L-3	35,438,065	99.57	98.20	44.03	91.74
10mg/L-1	34,776,253	99.68	98.54	44.06	92.38
10mg/L-2	34,485,579	99.67	98.59	44.09	92.04
10mg/L-3	38,168,206	99.65	98.5	44.08	92.22
25mg/L-1	35,308,587	99.58	98.25	44.09	91.60
25mg/L-2	37,159,907	99.60	98.33	44.20	90.23
25mg/L-3	39,149,536	99.52	97.97	44.10	91.74
100mg/L-1	34,104,901	99.63	98.33	44.01	92.71
100mg/L-2	34,490,058	99.61	98.33	44.02	92.75
100mg/L-3	36,212,357	99.73	98.79	43.90	93.19
